# High Serum Polyunsaturated Fatty Acids for the Increased Risk of Gestational Diabetes Mellitus Through LPC Pathways: A Prospective Nested Case–Control Study in China

**DOI:** 10.1155/jdr/3535965

**Published:** 2026-09-09

**Authors:** Shumin Zhao, Qingqing Meng, Xinli Xing, Junhong Leng, Weiqin Li, Cuiping Zhang, Jinnan Liu, Wen Yang, Juliana C. N. Chan, Gang Hu, Zhijie Yu, Xilin Yang, Jing Li

**Affiliations:** ^1^ Department of Epidemiology and Biostatistics, School of Public Health, Tianjin Medical University, Tianjin, China, tijmu.edu.cn; ^2^ Department of Obstetrics, Liaocheng Dongchangfu Maternal and Child Health Care Hospital, Shandong, China; ^3^ Tianjin Women and Children’s Health Center, Tianjin, China; ^4^ Nan Kai District Center for Disease Control and Prevention, Tianjin, China; ^5^ Department of Medicine and Therapeutics, Hong Kong Institute of Diabetes and Obesity and The Chinese University of Hong Kong-Prince of Wales Hospital-International Diabetes Federation Centre of Education, Hong Kong, China; ^6^ Chronic Disease Epidemiology Laboratory, Pennington Biomedical Research Center, Baton Rouge, Louisiana, USA, lsu.edu; ^7^ Population Cancer Research Program and Department of Pediatrics, Dalhousie University, Halifax, Canada, dal.ca; ^8^ Tianjin Center for International Collaborative Research on Environment, Nutrition and Public Health, Tianjin, China; ^9^ Tianjin Key Laboratory of Environment, Nutrition and Public Health, Tianjin, China

**Keywords:** arachidonic acid, docosahexaenoic acid, gestational diabetes mellitus, lysophosphatidylcholine, polyunsaturated fatty acid

## Abstract

**Aims:**

The study aimed to investigate the following: (1) whether early‐pregnancy serum polyunsaturated fatty acid (PUFA) species are associated with the risk of gestational diabetes mellitus (GDM); and (2) whether lysophosphatidylcholine (LPC)18:0 may statistically explain these associations in Chinese pregnant women.

**Materials and Methods:**

We conducted a 1:1 age‐matched case–control study of 486 pregnant women nested within a prospective cohort in Tianjin, China. Least Absolute Shrinkage and Selection Operator (LASSO) logistic regression analysis was used to identify the PUFA species independently associated with GDM. Conditional logistic regression was conducted to obtain odds ratios (ORs) and their 95% confidence intervals (CIs). Sobel mediation analysis was used to assess the potential statistical role of LPC18:0.

**Results:**

Among five PUFA species that differed significantly between cases and controls, arachidonic acid (AA, 20:4) and docosahexaenoic acid (DHA, 22:6) were selected and further analyzed. High AA (≥ 3.63 nmol/mL) and DHA (≥ 0.59 nmol/mL) were associated with increased GDM risk, with multivariable‐adjusted ORs of 2.89 (95% CI: 1.84–4.54) and 2.90 (95% CI: 1.82–4.63), respectively. These associations were stronger among women with pre‐pregnancy overweight/obesity (AA, OR: 5.32, 95% CI: 2.48–11.42; DHA, OR: 3.39, 95% CI: 1.65–6.99). Further adjustment for high LPC18:0 substantially attenuated the associations of AA (OR: 0.90, 95% CI: 0.45–1.80) and DHA (OR: 0.97, 95% CI: 0.51–1.85) with GDM risk, with Sobel test *p* values < 0.0001.

**Conclusions:**

Higher serum AA and DHA levels in early pregnancy were associated with increased GDM risk. LPC18:0 may statistically explain part of these observed associations.

## 1. Introduction

Gestational diabetes mellitus (GDM), a glucose metabolism disorder first recognized during pregnancy, affects 7%–25% of pregnancies worldwide [1, 2]. Its prevalence has increased over recent decades and GDM is associated with both short‐term obstetric complications and long‐term cardiometabolic risks in affected women and their offspring [3–5]. Evidence from intervention studies suggests that preventive strategies initiated early in pregnancy may be more effective than those started later [6]. Therefore, identifying early‐pregnancy metabolic alterations related to subsequent GDM risk may help improve understanding of GDM pathophysiology and support early risk assessment.

Polyunsaturated fatty acid (PUFA) species have distinct biological activities. For example, docosahexaenoic acid (DHA), a representative n‐3 PUFA, is generally involved in anti‐inflammatory and pro‐resolving processes, whereas arachidonic acid (AA), a representative n‐6 PUFA, can serve as a precursor for pro‐inflammatory lipid mediators [7]. However, several studies have reported that circulating levels of selected n‐3 and n‐6 PUFA species during pregnancy are higher in women with GDM or are associated with increased GDM risk [8–10]. These findings appear inconsistent with the conventional view that n‐3 PUFA species are metabolically protective. One possible explanation is that elevated circulating PUFA levels do not necessarily reflect enhanced biological activity or dietary intake, but may instead indicate altered lipid mobilization, PUFA utilization, or phospholipid remodeling in the context of early metabolic dysfunction.

GDM is characterized by insulin resistance, lipid metabolic disorder, and chronic low‐grade inflammation [11]. These metabolic features may influence circulating fatty acid profiles and phospholipid remodeling pathways. The Lands’ cycle is involved in phospholipid remodeling and may provide a biochemical link between PUFA release and lysophosphatidylcholine (LPC) generation [12, 13]. LPC species have also been implicated in inflammatory and metabolic disturbances, and our previous lipidomic study identified LPC18:0 as a GDM‐associated lipid mediator [14]. Based on this framework, LPC18:0 may help explain the association between altered circulating PUFA species and subsequent GDM risk.

In this nested case–control study, we aimed to investigate the following: (1) whether serum PUFA species in early pregnancy were associated with subsequent GDM risk; and (2) whether LPC18:0 may statistically explain the associations between selected serum PUFA species and GDM risk in Chinese pregnant women.

## 2. Materials and Methods

### 2.1. Population and Study Design

This nested case–control study was conducted within a population‐based prospective pregnancy cohort in six central districts of Tianjin, China. Details of the parent cohort have been reported previously [15]. Briefly, pregnant women were enrolled between October 2010 and August 2012 under the routine antenatal GDM screening program. Among 22,302 eligible cohort participants, 2991 provided fasting blood samples at their first prenatal care visit and were followed until GDM screening in mid‐pregnancy. The study was approved by the Clinical Research Ethics Committee of Tianjin Women and Children’s Health Center, and all participants provided written informed consent.

For the present analysis, 227 women without GDM screening records were excluded from the 2991 women with available early‐pregnancy fasting blood samples. Among the remaining 2764 women, 243 were diagnosed with GDM and included as cases. Controls were randomly selected from women who completed GDM screening and remained normoglycemic. Each case was matched to one control by maternal age within ±1 year, resulting in 243 case–control pairs and a final analytic sample of 486 women (Figure S1).

### 2.2. GDM Ascertainment and Covariate Assessment

GDM was ascertained using the standardized two‐step screening procedure implemented in the Tianjin prenatal care system. At 24–28 gestational weeks, all women underwent a non‐fasting 50‐g, 1‐h glucose challenge test (GCT) at local primary care facilities. Women with a plasma glucose (PG) concentration of ≥ 7.8 mmol/L were referred to the GDM clinic at Tianjin Women and Children’s Health Center for a 75‐g, 2‐h oral glucose tolerance test (OGTT) after an overnight fast of at least 8 h. GDM was diagnosed according to the International Association of Diabetes and Pregnancy Study Groups criteria if any of the following thresholds were met: fasting PG ≥ 5.1 mmol/L, 1‐h PG ≥ 10.0 mmol/L, or 2‐h PG ≥ 8.5 mmol/L [16].

Maternal anthropometric and clinical measurements were obtained at the first prenatal care visit by trained nurses or obstetricians using standardized procedures. Height, weight, systolic blood pressure (SBP), and diastolic blood pressure (DBP) were recorded at baseline. Because weight gain is generally limited in early pregnancy, weight measured at the first prenatal visit was used as an approximation of pre‐pregnancy weight to calculate pre‐pregnancy body mass index (BMI) [17]. Maternal weight was measured again at the time of the GCT. Information on maternal age, family history of diabetes in first‐degree relatives, parity, ethnicity, educational attainment, smoking, and alcohol consumption before or during pregnancy was collected using structured questionnaires during prenatal care.

### 2.3. Measurement of Serum PUFA Species and LPC18:0

Fasting serum samples collected in early pregnancy were used to quantify PUFA species and LPC18:0. Targeted lipid profiling was performed using liquid chromatography‐tandem mass spectrometry (LC‐MS/MS). Chromatographic separation was conducted on an AB SCIEX TripleTOF 5600 mass spectrometer coupled with an Eksigent Ultra LC 100 system. A Waters XBridge Peptide BEH C18 column (2.1 × 100 mm, 3.5 *μ*m), equipped with a Phenomenex C18 pre‐column (4 × 2.0 mm), was used for separation.

Targeted PUFA species were identified by matching chromatographic peaks against an authentic free fatty acid standard database in PeakView 1.2. Absolute concentrations were calculated in MultiQuant 2.1 using calibration curves adjusted by specific internal standards. The detected PUFA species included FFA18:2, FFA20:2, FFA20:4, FFA20:5, and FFA22:6. LPC18:0 was quantified using an LC‐MS/MS method described previously [14].

### 2.4. Statistical Analysis

Statistical analyses were performed using SAS version 9.4 (SAS Institute Inc., Cary, NC, United States) and R software version 4.4.1 (R Foundation for Statistical Computing, Vienna, Austria). Continuous variables were expressed as means ± standard deviation (SD) or median (interquartile range), and categorical variables were presented as frequencies (%). Baseline characteristics were compared between matched case–control pairs using paired Student’s *t*‐test or Wilcoxon signed‐rank test for the continuous variables, and McNemar test or Fisher’s exact test for categorical variables, as appropriate.

Least Absolute Shrinkage and Selection Operator (LASSO) logistic regression was used to identify PUFA species most robustly associated with GDM while reducing collinearity among candidate variables [18]. The analysis was performed using the “glmnet” package in R, with 10‐fold cross‐validation used to select the optimal tuning parameter. Candidate variables included the five serum PUFA species, pre‐pregnancy BMI, SBP, Han nationality, family history of diabetes in first‐degree relatives, education, weight gain to GCT, smoking before or during pregnancy, alcohol consumption before or during pregnancy, and alanine aminotransferase. Because cases and controls were matched by age within ±1 year, age at the first prenatal care visit was forced into the model as a fixed effect [18].

PUFA species selected by the LASSO model were further examined using restricted cubic spline (RCS) analysis to assess potential nonlinear associations with GDM risk. These PUFA species were then dichotomized at their median values for subsequent analyses. Conditional logistic regression models were used to estimate odds ratios (ORs) and 95% confidence intervals (CIs) for the associations between selected PUFA species and GDM risk. Crude models were first fitted, followed by multivariable models adjusted for the covariates included in the LASSO model, except for age because of the matched design.

Stratified analyses were performed according to pre‐pregnancy BMI using 24 kg/m^2^ as the cutoff based on the Chinese criterion for overweight [19]: non‐overweight, < 24 kg/m^2^; and overweight/obesity, ≥ 24 kg/m^2^. Overweight and obesity were combined because of the limited number of women with obesity in this nested case–control sample, which could otherwise have led to unstable estimates.

Given the previously reported association between LPC18:0 and GDM risk in this cohort [14], Spearman correlation analysis was used to evaluate the correlations between selected PUFA species and LPC18:0. LPC18:0 was dichotomized at the median value, and additional models were fitted to assess whether adjustment for high LPC18:0 attenuated the associations between selected PUFA species and GDM risk. Sobel tests were further used to evaluate the statistical mediation effect of LPC18:0 [20]. A two‐sided *p* < 0.05 was considered statistically significant.

## 3. Results

### 3.1. Clinical Characteristics in Pregnant Women

A total of 486 pregnant women were recruited in this study. The mean age of women was 29.2 ± 3.1 years and the median gestational age was 10.0 (9.0–11.0) weeks at enrollment. Compared with the controls, women with GDM had higher weight, pre‐pregnancy BMI, SBP, DBP, alanine aminotransferase, LPC18:0 and GCT glucose levels. Besides, they were more likely to have family history of diabetes in first‐degree relatives (Table S1).

### 3.2. Characteristics of Serum PUFA Species in Pregnant Women

The concentrations of all the detected PUFA species, namely FFA18:2, FFA20:2, FFA20:4, FFA20:5, and FFA22:6, were higher in GDM subgroups versus controls, and the differences of all PUFA concentrations between all GDM group and controls were statistically significant (Table 1).

**Table 1:** Characteristics of serum PUFA species in GDM and non‐GDM women.

	Non‐GDM (*N* = 243)	GDM subgroup^a^			All GDM (*N* = 243)	*p*value^§^
		FPG (*N* = 141)	1 h‐PG (*N* = 67)	2 h‐PG (*N* = 35)		
FFA18:2, nmol/mL	105.6 (68.6–151.5)	129.0 (87.4–183.1)^‡^	159.1 (96.7–205.1)^‡^	126.2 (95.6–168.9)^†^	130.8 (90.5–188.7)	< 0.0001
FFA20:2, nmol/mL	1.99 (1.58–2.44)	2.27 (1.82–2.85)^‡^	2.17 (1.84–2.87)^†^	2.08 (1.89–2.84)^†^	2.21 (1.83–2.87)	0.0004
FFA20:4, nmol/mL	3.14 (2.16–4.27)	4.07 (3.00–5.58)^‡^	4.27 (3.00–5.62)^‡^	4.17 (2.94–6.21)^†^	4.17 (3.00–5.61)	< 0.0001
FFA20:5, nmol/mL	0.31 (0.23–0.45)	0.47 (0.33–0.64)^‡^	0.41 (0.33–0.58)^‡^	0.45 (0.32–0.79)^‡^	0.46 (0.33–0.64)	< 0.0001
FFA22:6, nmol/mL	0.51 (0.34–0.68)	0.64 (0.48–0.86)^‡^	0.68 (0.50–1.07)^‡^	0.71 (0.47–0.98)^‡^	0.67 (0.49–0.90)	< 0.0001

*Note:* Data are reported in median (25th–75th percentile).

Abbreviations: FFAs, free fatty acids; FPG, fasting plasma glucose; GDM, gestational diabetes mellitus; IADPSG, International association of diabetes and pregnancy study groups; PG, plasma glucose; PUFA, polyunsaturated fatty acid.

^a^Based on IADPSG′s criteria: FPG ≥ 5.1 mmol/L, the 1‐h PG ≥ 10.0 mmol/L, or the 2‐h PG ≥ 8.5 mmol/L.

^†^ *p* value < 0.05 between non‐GDM group and GDM subgroups detected by the Wilcoxon rank‐sum test.

^‡^ *p* value < 0.001 between non‐GDM group and GDM subgroups detected by the Wilcoxon rank‐sum test.

^§^ *p* values were obtained using the Wilcoxon signed‐rank test to compare the non‐GDM group with the all GDM group.

### 3.3. Association of FFA20:4 and FFA22:6 With GDM

**Figure 1:**
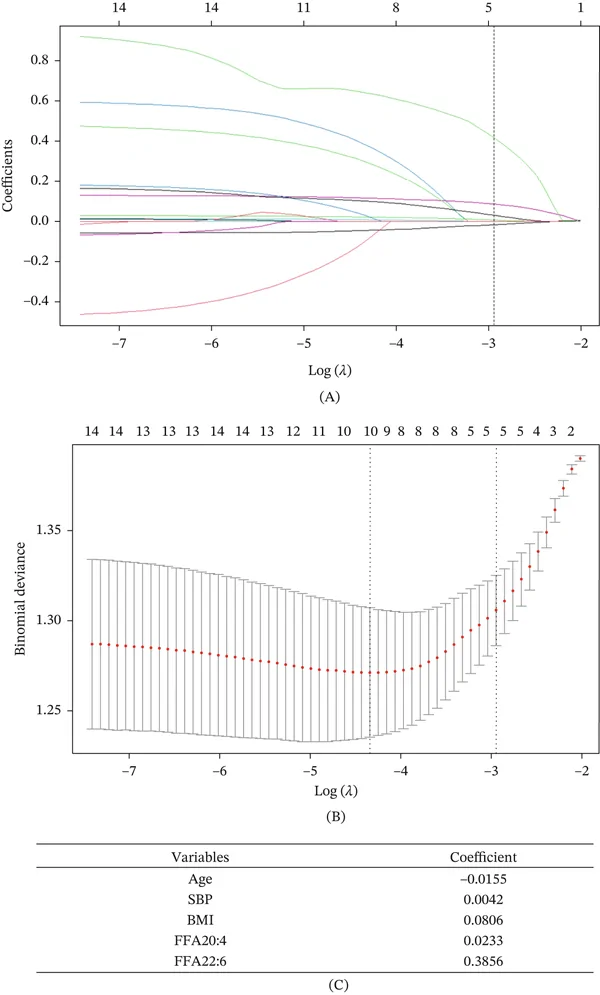
Screening of serum PUFA species based on LASSO logistic regression. (A) The variation characteristics of the coefficient of PUFA species; (B) the process of selecting parameter *λ* in LASSO logistic regression model by 10‐fold cross validation method; (C) the coefficients of variables selected by LASSO logistic regression. Abbreviations: BMI, body mass index; FFA, free fatty acid; PUFA, polyunsaturated fatty acid; SBP, systolic blood pressure.

The results of LASSO logistic regression analysis were shown in Figure 1. The variation characteristics of the coefficients were presented in Figure 1A. The most optimal option for analysis was the inclusion of two PUFA species into models, as illustrated in Figure 1B. When the tuning parameter *λ* = 0.0523 (Log *λ* = −2.95), FFA20:4 and FFA22:6 were selected, and the corresponding coefficients were illustrated in Figure 1C.

In RCS analysis, FFA20:4 and FFA22:6 were both significant nonlinear positive associations with the risk of GDM (*p* of nonlinear < 0.001) (Figure S2). In univariate analysis, high FFA20:4 (FFA20:4 ≥ 3.63 nmol/mL) and FFA22:6 (FFA22:6 ≥ 0.59 nmol/mL) were both associated with increased GDM risk. Specifically, when comparing high FFA20:4 level with low FFA20:4 level, the crude OR was 2.76 (95% CI: 1.85–4.11). Similarly, when comparing high FFA22:6 level with low FFA22:6 level, the crude OR was 2.84 (95% CI: 1.89–4.28). In multivariate model, following the adjustment for other covariates, the ORs for the associations between high vs. low levels of FFA20:4 and FFA22:6 and GDM were 2.89 (95% CI: 1.84–4.54) and 2.90 (95% CI: 1.82–4.63), respectively. Among women with high pre‐pregnancy BMI, the adjusted ORs for high FFA20:4 and FFA22:6 in the relation to GDM increased to 5.32 (95% CI: 2.48–11.42) and 3.39 (95% CI: 1.65–6.99), respectively (Table 2).

**Table 2:** Odds ratios of serum PUFA species for the risk of GDM.

	OR	95% CI	*p*value
Univariable model			
FFA20:4, per SD	1.96	1.49–2.56	< 0.0001
FFA22:6, per SD	1.92	1.47–2.50	< 0.0001
FFA20:4 ≥ 3.63 versus < 3.63 nmol/mL	2.76	1.85–4.11	< 0.0001
FFA22:6 ≥ 0.59 versus < 0.59 nmol/mL	2.84	1.89–4.28	< 0.0001
Multivariable model			
FFA20:4, per SD	1.90	1.41–2.55	< 0.0001
FFA22:6, per SD	1.77	1.35–2.33	< 0.0001
FFA20:4 ≥ 3.63 versus < 3.63 nmol/mL	2.89	1.84–4.54	< 0.0001
FFA22:6 ≥ 0.59 versus < 0.59 nmol/mL	2.90	1.82–4.63	< 0.0001
Among pregnant women with pre‐pregnancy BMI < 24.0 kg/m^2a^			
FFA20:4, per SD	1.87	1.34–2.62	0.0003
FFA22:6, per SD	1.85	1.35–2.54	0.0001
FFA20:4 ≥ 3.63 versus < 3.63 nmol/mL	2.03	1.26–3.26	0.0036
FFA22:6 ≥ 0.59 versus < 0.59 nmol/mL	2.23	1.38–3.58	0.0010
Among pregnant women with pre‐pregnancy BMI ≥24.0 kg/m^2a^			
FFA20:4, per SD	1.83	1.14–2.95	0.0127
FFA22:6, per SD	1.46	0.99–2.16	0.0571
FFA20:4 ≥ 3.63 versus < 3.63 nmol/mL	5.32	2.48–11.42	< 0.0001
FFA22:6 ≥ 0.59 versus < 0.59 nmol/mL	3.39	1.65–6.99	0.0009

*Note:* Multivariable model: Adjusted for adjusted for systolic blood pressure, Han nationality, family history of diabetes in the first‐degree relatives, education, current smoking before or during pregnancy, alcohol drinking before or during pregnancy, weight gain to glucose challenge test, alanine aminotransferase.

Abbreviations: BMI, body mass index; CI, confidence interval; FFA, free fatty acid; GDM, gestational diabetes mellitus; OR, odds ratio; PUFA, polyunsaturated fatty acid.

^a^Variables listed in multivariable model were also adjusted in stratified analyses.

### 3.4. Mediation Effects of LPC18:0 for FFA20:4 and FFA22:6 on GDM Risk

LPC18:0 was moderately correlated with both AA and DHA (Table S2). In mediation analysis, both FFA20:4 and FFA22:6 were significantly associated with high LPC18:0 (OR: 6.31, 95% CI: 4.11–9.70; OR: 4.90, 95% CI: 3.24–7.43, respectively). High LPC18:0 was strongly associated with increased GDM risk (OR: 17.05, 95% CI: 7.72–37.66; OR: 16.47, 95% CI: 7.59–35.76). After adjusting for high LPC18:0, the direct associations between FFA20:4 or FFA22:6 and GDM risk were no longer significant (*p* > 0.05), with a significant mediation effect (Sobel test *p* < 0.0001) (Table 3).

**Table 3:** ORs and 95% CIs for the mediation effects of LPC18:0 and the risk of GDM.

	*β* (SD)	OR (95% CI)	*p*value
**Mediation effect of high LPC18:0 for high FFA20:4 to increased risk of GDM**			
Model A (LPC18:0 > 18.00 nmol/mL as the outcome)			
FFA20:4 ≥ 3.63 nmol/mL	1.84 (0.22)	6.31 (4.11‐9.70)	< 0.0001
Model B (GDM as the outcome)			
LPC18:0 ≥ 18.00 nmol/mL	2.83 (0.40)	17.05 (7.72‐37.66)	< 0.0001
Model C (GDM as the outcome)			
FFA20:4 ≥ 3.63 nmol/mL	‐0.11 (0.36)	0.90 (0.45‐1.80)	0.7602
Sobel test for mediation effect			< 0.0001
**Mediation effect of high LPC18:0 for high FFA22:6 to increased risk of GDM**			
Model A (LPC18:0 > 18.00 nmol/mL as the outcome)			
FFA22:6 ≥ 0.59 nmol/mL	1.59 (0.21)	4.90 (3.24‐7.43)	< 0.0001
Model B (GDM as the outcome)			
LPC18:0 ≥ 18.00 nmol/mL	2.80 (0.40)	16.47 (7.59‐35.76)	< 0.0001
Model C (GDM as the outcome)			
FFA22:6 ≥ 0.59 nmol/mL	‐0.03 (0.33)	0.97 (0.51‐1.85)	0.9272
Sobel test for mediation effect			< 0.0001

*Note:* Model A was adjusted for systolic blood pressure, Han nationality, family history of diabetes in the first‐degree relatives, education, current smoking before or during pregnancy, alcohol drinking before or during pregnancy, weight gain to glucose challenge test, alanine aminotransferase, and pre‐pregnancy body mass index. Model B was adjusted for the variables listed in Model A. Model C was further adjusted for LPC18 : 0 ≥ 18.00 nmol/mL in addition to the variables listed in Model A.

Abbreviations: CI, confidence interval; FFA, free fatty acid; GDM, gestational diabetes mellitus; LPC, lysophosphatidylcholine; OR, odds ratio.

## 4. Discussion

In this nested case–control study, we found that women who subsequently developed GDM had elevated serum PUFA levels in early pregnancy. Among the measured PUFA species, FFA20:4 (AA) and FFA22:6 (DHA) were selected by the LASSO model and were associated with increased GDM risk. These associations appeared stronger among women with higher pre‐pregnancy BMI. After further adjustment for high LPC18:0, the associations of AA and DHA with GDM risk were substantially attenuated and no longer statistically significant. These findings suggest that LPC18:0 may statistically explain part of the observed associations between selected serum PUFA species and GDM risk.

PUFA plays an important role in metabolism during pregnancy and fetal development, and may also be related to GDM through pathways involving insulin sensitivity, lipid metabolism, and inflammatory responses. Several population‐based studies have examined circulating PUFA levels in relation to GDM risk, although their findings appear complex. Higher circulating PUFA levels have been observed in women with GDM compared with controls. For example, a prospective cohort study from Iceland reported that pregnant women with GDM had elevated plasma PUFA levels in the first trimester [8]. A nested case–control study from China similarly showed that women who developed GDM had higher early‐pregnancy plasma PUFA levels than controls [9]. Meta‐analyses based mainly on mid‐to‐late pregnancy samples further reported that the percentages of AA, DHA, and n‐6 PUFA in plasma or serum total fatty acids were higher in women with GDM [21]. A US case–control study also reported a graded increase in several fatty acids across control, impaired glucose tolerance, and GDM groups in the third trimester, suggesting a potential correlation between glycemic dysregulation and PUFA levels [22]. Consistent with these studies, our results showed that serum PUFA levels, particularly AA and DHA, were higher in early pregnancy among women who later developed GDM. This finding is notable because AA and DHA have divergent biological roles.

AA, a representative n‐6 PUFA, can serve as a precursor for eicosanoids and other lipid mediators involved in inflammatory responses [7], whereas DHA, a representative n‐3 PUFA, is generally involved in anti‐inflammatory and pro‐resolving processes [7]. However, circulating concentrations of PUFA species do not necessarily reflect their biological activity or functional effects. In this context, elevated serum DHA in early pregnancy may be better understood as a marker of altered lipid mobilization, fatty acid utilization, or phospholipid remodeling in women at higher metabolic risk, rather than indicating a direct pathogenic role of DHA per se [23, 24]. This interpretation is consistent with the stronger associations observed among women with higher pre‐pregnancy BMI, a group more likely to have insulin resistance, chronic low‐grade inflammation, and lipid metabolic disturbance. Taken together, these findings suggest that the simultaneous elevation of AA and DHA may represent a broader metabolic imbalance rather than a uniform biological effect of different PUFA species.

A possible explanation for the statistical role of LPC18:0 is related to phospholipid remodeling. The Lands’ cycle is an important pathway for phosphatidylcholine remodeling, involving phospholipid deacylation and reacylation reactions [12, 13]. During the deacylation step, phospholipase A2 can hydrolyze fatty acyl chains from phosphatidylcholine, leading to the release of free fatty acids, including PUFA species such as AA and DHA, and the generation of LPC [12]. LPC can subsequently be reacylated by LPC acyltransferases to regenerate phosphatidylcholine [12, 13]. Therefore, correlated changes in serum PUFA species and LPC18:0 may reflect alterations in phospholipid remodeling. LPC species have also been implicated in inflammatory and metabolic disturbances, and our previous lipidomic study identified LPC18:0 as a GDM‐associated inflammatory lipid mediator [14]. In the present study, AA and DHA were correlated with LPC18:0, and their associations with GDM risk were markedly attenuated after adjustment for LPC18:0. These results suggest that LPC18:0 may represent a lipid‐related inflammatory or metabolic marker that helps explain the observed associations between selected circulating PUFA species and GDM risk. However, because we did not directly measure phospholipase activity, LPC acyltransferase activity, PUFA metabolic flux, DHA‐derived specialized pro‐resolving mediators, or downstream inflammatory cytokines, this proposed pathway remains hypothetical and requires further experimental validation.

Our study has potential public health and clinical implications. Although PUFA species are conventionally considered important for maternal metabolism and fetal development, elevated circulating PUFA levels in early pregnancy may also serve as biomarkers of metabolic disturbance in women at increased risk of GDM. In particular, the stronger associations observed among women with higher pre‐pregnancy BMI suggest that combined assessment of adiposity status and circulating lipid profiles may help identify women at higher GDM risk. Since circulating serum PUFA species and dietary PUFA intake represent related but distinct exposure indicators, our findings do not directly address the potential effects of PUFA supplementation during pregnancy. Circulating PUFA concentrations may reflect dietary intake to some extent, but they are also influenced by endogenous fatty acid metabolism, lipid mobilization, phospholipid remodeling, inflammation, and pregnancy‐related metabolic changes [25]. Further studies integrating dietary assessment, longitudinal lipidomics, and mechanistic biomarkers are needed to clarify how dietary PUFA intake, circulating PUFA profiles, phospholipid remodeling, LPC metabolism, and GDM development are related.

Several limitations should be acknowledged. First, the study population was relatively homogeneous, with limited geographic and ethnic diversity, although our findings were generally consistent with several previous studies. Further validation in diverse populations is needed. Second, dietary and lifestyle factors were not comprehensively assessed, which may have introduced residual confounding. Third, we were unable to establish a threshold value for serum PUFA levels, especially DHA, which could indicate disturbances in DHA metabolism or phospholipid remodeling. Fourth, although serum PUFA levels may sensitively reflect metabolic changes, they do not fully capture the functional roles of PUFA incorporated into complex lipids or membrane phospholipids, which may have different pathophysiological implications for GDM. Fifth, PUFA species and LPC18:0 were measured in the same early‐pregnancy serum samples. Although blood samples were collected before GDM diagnosis, the temporal sequence between PUFA alterations and LPC18:0 cannot be definitively established. Therefore, the mediation analysis should be interpreted as statistical mediation rather than confirmation of a causal pathway from PUFA alterations to LPC18:0 and subsequent GDM. Finally, our mechanistic interpretations remain hypothesis‐generating because we did not directly measure key enzymes, lipid metabolic flux, or inflammatory mediators involved in phospholipid remodeling. Longitudinal metabolomic studies and experimental investigations are needed to clarify the biological relationships among PUFA species, LPC18:0, and GDM development.

## 5. Conclusions

In conclusion, in this nested case–control study, higher serum PUFA levels in early pregnancy, particularly AA and DHA, were associated with increased GDM risk. These associations were more pronounced among women with higher pre‐pregnancy BMI. Further adjustment for LPC18:0 substantially attenuated the associations of AA and DHA with GDM risk, suggesting that LPC18:0 may statistically explain part of these observed associations. Further longitudinal and experimental studies are needed to validate these findings and clarify the underlying biological mechanisms.

## Author Contributions

Conceptualization, methodology and supervision: X.Y. and J.L. Data curation and investigation: J.H.L., W.L., and C.Z. Formal analysis: J.N.L., Q.M., and W.Y. Validation: S.Z. and Q.M. Visualization: S.Z., Q.M., and X.X. Writing – original draft: S.Z., Q.M., and X.X. Writing – review and editing: J.C.N.C., G.H., Z.Y., X.Y. and J.L. Funding acquisition, resource and project administration: J.L. All authors contributed to the critical revision of the manuscript for important intellectual content. The corresponding authors had full access to all the data in the study and had the final responsibility to submit for publication. S.Z., Q.M., and X.X. have equally contributed to the manuscript.

## Funding

This study was supported by the National Natural Science Foundation of China (10.13039/501100001809; Grant No. 82200932) and the Science Foundation of Liaocheng Dongchangfu Maternal and Child Health Care Hospital (Grant Nos. DCFY‐KLT‐2023‐02 and DCFY‐KLT‐2023‐06).

## Disclosure

All authors have read and approved the final version of the manuscript. X.Y. & J.L. (the corresponding authors) had full access to all of the data in this study and takes complete responsibility for the integrity of the data and the accuracy of the data analysis.

## Ethics Statement

Ethics approval was obtained from the Ethics Committee for Clinical Research of Tianjin Women and Children’s Health Center (ref. no. 2009‐02).

## Conflicts of Interest

The authors declare no conflicts of interest.

## Supporting information


**Supporting Information** Additional supporting information can be found online in the Supporting Information section. Additional figures (Figures S1 to S2) and tables (Tables S1 to S2) are provided in the Supporting information.

## Data Availability

The datasets generated during and/or analyzed during the current study are available from the corresponding author upon reasonable request.
