# Supplementary material for: High Serum Polyunsaturated Fatty Acids for the Increased Risk of Gestational Diabetes Mellitus Through LPC Pathways: A Prospective Nested Case–Control Study in China
Source: J Diabetes Res. 2026 Sep 9;2026:3535965. doi: 10.1155/jdr/3535965 (PMC13556162; doi:10.1155/jdr/3535965)
Supplement: Supplementary file 1 [file JDR-2026-3535965-s001.pdf]

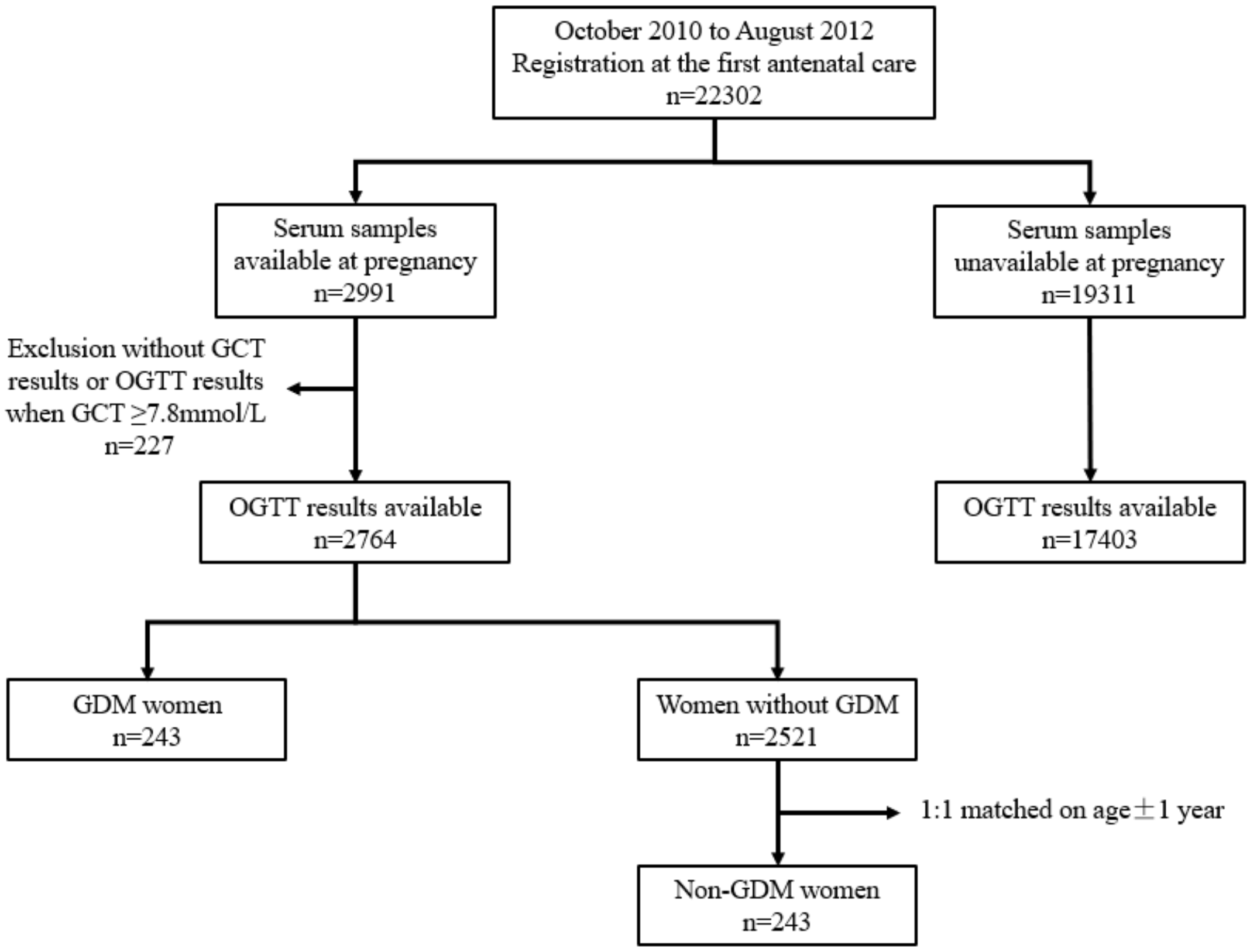

Supplementary Fig. 1—Flow diagram for population selection within the nested case-control study

Abbreviations: GCT, glucose challenge test; OGTT, oral glucose tolerance test; GDM, gestational diabetes mellitus.

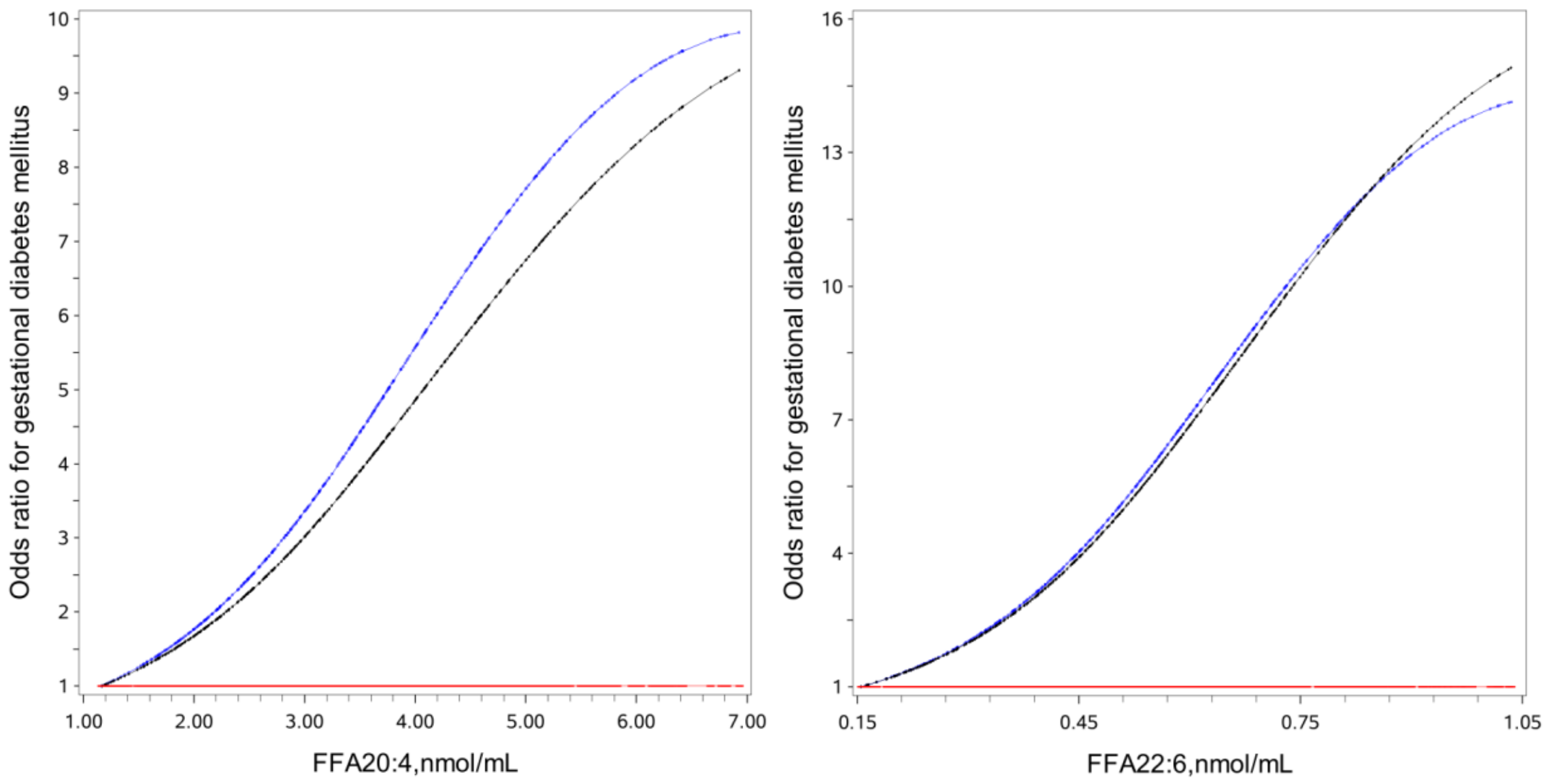

Supplementary Fig. 2—Associations between serum PUFA levels and the risk of GDM

Length: The black (crossed) lines were derived from univariable analyses, whereas blue (dot) lines were derived from multivariable analyses, adjusted for pre-pregnancy body mass index, systolic blood pressure, Han nationality, family history of diabetes in the first-degree relatives, education, current smoking before or during pregnancy, alcohol drinking before or during pregnancy, weight gain to glucose challenge test, alanine aminotransferase, and the red (straight) lines were the reference line at OR=1.

Abbreviations: GDM, gestational diabetes mellitus; PUFA, polyunsaturated fatty acid; FFA, free fatty acid.

Supplementary Table 1—Clinical and biochemical characteristics of GDM and non-GDM women

Characteristic	Non-GDM (N=243)	GDM (N=243)	*P*-value
**Variables at registration**			
Age, year	29.2±3.3	29.2±2.7	0.2962*
Height, cm	163.2±4.6	163.1±5.0	0.9500*
Weight, kg	58.2±9.6	63.7±10.5	<0.0001*
BMI, kg/m^2	21.8±3.4	23.9±3.6	<0.0001*
BMI in category			<0.0001†
<24.0 kg/m^2	186 (76.5)	135 (55.5)	
≥24.0-< 28.0 kg/m^2	45 (18.5)	77 (31.7)	
≥28.0 kg/m^2	12 (5.0)	31 (12.8)	
Gestational age, week	10.1±2.0	10.1±2.1	0.7976*
Systolic blood pressure, mmHg	104.0±10.5	108.3±10.5	<0.0001*
Diastolic blood pressure, mmHg	67.9±7.7	70.6±8.0	0.0001*
Han nationality	234 (96.3)	238 (97.9)	0.2850†
Education >12 years	132 (54.3)	135 (55.6)	0.7797†
Parity ≥1	12 (4.9)	14 (5.8)	0.6831†
Family history of diabetes in the first-degree relatives	14 (5.8)	30 (12.4)	0.0114†
Current smoker before pregnancy	13 (7.7)	15 (7.0)	0.6698†
Alcohol drinker before pregnancy	57 (23.5)	72 (29.6)	0.1198†
Alanine aminotransferase, U/L	16.0 (10.7-21.0)	19.0 (14.0-26.0)	0.0001*
**Serum metabolites**			

LPC18:0, nmol/mL	13.1 (10.8-17.2)	22.8 (19.0-28.9)	<0.0001[*]
**Variables during pregnancy**			
Current smoker during pregnancy	1 (0.4)	2 (0.8)	0.5637[†]
Alcohol drinker during pregnancy	2 (0.8)	2 (0.8)	1.0000[†]
Gestational week at GCT, week	25.2±2.2	24.9±1.4	0.1118[*]
Weight gain up to the time of GCT, kg	8.7±3.2	8.4±3.6	0.3004[*]
GCT glucose, mmol/L	6.3 (5.4-7.2)	9.0 (8.4-10.0)	<0.0001[*]

Data are reported in mean ± SD or median (25th-75th percentile) or number (percentages).

* Derived from paired t-test or Wilcoxon signed-rank test.

† Derived from McNemar test or Fisher's exact test.

Abbreviations: GDM, gestational diabetes mellitus; BMI, body mass index; LPC, lysophosphatidylcholine; GCT, glucose challenge test.

Supplementary Table 2—Spearman correlation coefficients of serum PUFA species for LPC18:0

	Correlation Coefficient	*P*-value
**All pregnant women (n=486)**		
FFA20:4	0.5600	<0.0001
FFA22:6	0.4806	<0.0001
**Women with pre-pregnancy BMI <24.0 kg/m^2 (n=165)**		
FFA20:4	0.5369	<0.0001
FFA22:6	0.4472	<0.0001
**Women with pre-pregnancy BMI ≥24.0 kg/m^2 (n=321)**		
FFA20:4	0.5592	<0.0001
FFA22:6	0.5016	<0.0001

Abbreviations: PUFA, polyunsaturated fatty acid; FFA, free fatty acid; LPC, lysophosphatidylcholine; BMI, body mass index.
